# A serial 3- and 9-year optical coherence tomography assessment of vascular healing response to sirolimus- and paclitaxel-eluting stents

**DOI:** 10.1007/s10554-018-1437-7

**Published:** 2018-08-30

**Authors:** Mariusz Tomaniak, Łukasz Kołtowski, Arkadiusz Pietrasik, Adam Rdzanek, Jacek Jąkała, Klaudia Proniewska, Krzysztof Malinowski, Tomasz Mazurek, Krzysztof J. Filipiak, Salvatore Brugaletta, Grzegorz Opolski, Janusz Kochman

**Affiliations:** 1 First Department of Cardiology, Medical University of Warsaw, ul. Banacha 1a, 02-097 Warsaw, Poland; 2 Krakow Cardiovascular Research Institute, Krakow, Poland; 3 Institut Clinic Cardiovascular, IDIBAPS, Hospital Clinic, University of Barcelona, Barcelona, Spain

**Keywords:** Optical coherence tomography, Early-generation drug-eluting stent, Healing response, Late catch up

## Abstract

Early-generation drug-eluting stents (DES) have been demonstrated to delay vascular healing. Limited optical coherence tomography (OCT) data on the very long-term neointimal response after DES implantation are available. The aim of this study was a serial OCT assessment of neointimal thickness, stent strut coverage, malapposition, and protrusion as markers of neointimal response at 3 and 9 years after implantation of sirolimus-eluting stents (SES) and paclitaxel-eluting stents (PES). In this single-centre, longitudinal study consecutive patients undergoing elective PCI with SES or PES were included. OCT analysis was performed after 3 and 9 years by the independent core laboratory. A total of 22 subjects (8 SES and 14 PES) underwent an OCT assessment at 3 and 9 years post index procedure. The lumen, neointimal and malapposition area and the neointimal thickness (SES ∆50 µm, p = 0.195, PES ∆10 µm, p = 0.951) did not change significantly over the 6 year follow-up. No differences in the incidence of uncovered, malapposed or protruding struts were found in each type of stent. At 3 and 9 years after PCI, implantation of early-generation SES and PES may be associated with similar neointimal thickness, strut coverage, malapposition and protrusion, as assessed by serial OCT examination among patients with uneventful follow-up at 3 years post procedure. The small size of the study warrants judicious interpretation of our results and confirmation in larger multimodality imaging studies, including patients treated with contemporary stent platforms.

**Electronic supplementary material:**

The online version of this article (10.1007/s10554-018-1437-7) contains supplementary material, which is available to authorized users.

## Introduction

Late adverse events such as stent thrombosis and in-stent restenosis have been recognized as major concerns after early-generation drug-eluting stents (DES) implantation [1–6]. Among the varied factors related to these complications, the most important encompass delayed vascular healing with impaired strut coverage and strut malapposition [1, 2, 5, 7–9].

Incomplete neointimal coverage of stent struts leading to their direct exposition to the blood flow was identified as a strong surrogate indicator of endothelialization and a significant morphometric predictor of late stent thrombosis (ST) [10, 11]. On the other hand, autopsy studies of early generation DES demonstrated features of continuous neointimal growth at the extended follow-up, referred to as *late catch-up* phenomenon [2, 7, 12, 13], thus prompting the need for long-term evaluation of vessel healing response to this polymer-coated metallic devices. The longest systematic follow-ups in autopsy studies evaluating neointimal growth after DES implantation reached up to maximally 6 years after PCI [2, 13].

At present, optical coherence tomography (OCT) appears the most precise intravascular diagnostic method enabling in vivo identification of incomplete stent strut coverage, strut malapposition, protrusion and presence of intraluminal thrombi [14, 15]. Still, limited OCT data are available on the very long-term neointimal response after DES implantation, with the longest follow-ups reported up to 5 years [9, 16–19]. Within this time frames, both sirolimus eluting stents (SES) and paclitaxel-eluting stents (PES) incidentally presented delayed arterial healing, including unfavourable strut outcome in terms of neointimal coverage and interaction with the vessel wall [12, 16, 20].

Notably, although the early generation DES are no longer widely used in clinical practice, they have been already implanted in millions of patients over the past decades. We hypothesized that despite uneventful early- and mid-term follow up, the vessel wall may be subject to dynamic morphometric changes at the extended observation, resultant from prosthesis-related inflammatory processes and toxicity.

Given this background, we performed a serial, very long-term 3- and 9-year OCT assessment of stent strut coverage, malapposition and protrusion as markers of vascular healing response to SES and PES implanted in patients with stable coronary artery disease (SCAD).

## Methods

### Study population

This is a single-centre, non-randomized, longitudinal study that analysed consecutive patients presenting with SCAD patients who underwent elective PCI with implantation of either SES (Cypher, Cordis) or PES (Taxus, Boston Scientific) between January 2003 and December 2004 and underwent the long-term OCT evaluation.

The inclusion criteria involved: single DES implantation in clinically and angiographically relevant stenosis of a native vessel, implantation of a stent within 2.5–3.5 mm in diameter, at least 36 months of uneventful follow-up after the index PCI, informed consent to participate in the intravascular imaging follow-up, adherent dual antiplatelet therapy continuation for the 12 months following the index procedure, according to the recommendations applicable at the time when the study was conducted.

Exclusion criteria comprised a history of target vessel revascularization (TVR), myocardial infarction (MI) and stroke in a period between index PCI and planned OCT examination; left main as a culprit vessel, lesions located < 10 mm from the native vessel ostium due to lack of possibility to perform OCT measurement with proximal balloon occlusion, PCI of a chronic total occlusion of a native artery, chronic kidney disease with baseline estimated glomerular filtration rate (GFR) < 30 mL/min/1.73 m^2^. All index procedures were performed using routine interventional cardiology techniques, with performance of predilatation and post-dilatation left to the operator’s discretion.

After inclusion, OCT examination was perfomed at ≥ 3 years following the initial procedure. Thereafter, patients were clinically followed-up for up to 9 years and underwent second OCT evaluation at the end of the observation period.

### Optical coherence tomography imaging

OCT images at 3 year follow-up were acquired using a time-domain OCT imaging system (M2 system, LightLab Imagining Inc., Westford, Massachusetts, US) in accordance with manufacturer recommendations. Briefly, the occlusive over-the-wire balloon (Helios) was advanced in the target vessel proximally to the lesion. An optic fibre probe (ImageWire, LightLab Imaging Inc.) was inserted through the balloon to the distal part of the vessel. After proximal balloon occlusion an automatic OCT pullback was performed with a continuous Ringer lactate solution infusion.

At 9 years, OCT images were obtained with a commercially available frequency domain OCT imaging system (C7XRsystem) with Dragonfly^®^ image catheters, (LightLab Imaging Inc., Westford, Massachusetts, US) using the non-occlusive flushing technique. Following the diagnostic coronary angiography, the ImageWire (Lightlab Imaging) was carefully placed distal to the stenosis. After administration of 200 mg of intra-coronary nitroglycerin, the target vessel was flushed via the guiding catheter with isomolar, nonionic contrast liquid.

All OCT imaging analyses have been performed offline by the same independent core laboratory [Krakow Cardiovascular Research Institute (KCRI), Krakow, Poland] using the proprietary software from LightLab Imaging by two analysts blinded to the angiographic data and patients’ clinical characteristics. Stent analyses for strut coverage, apposition and protrusion at frames were conducted at 1 mm longitudinal intervals, based on the methodology applied in previous studies using OCT evaluation of DES [9, 14, 15, 19, 21, 22]. The landmarks such as stent edge, side branches and calcifications were used to match the corresponding cross sections between the 3- and 9-year examinations for evaluation of changes in lumen area, minimal lumen area (MLA), neointimal thickness as well as the percentage of uncovered, malapposed and protruding struts. Struts located at the ostium of side branches, with no vessel wall behind, were excluded from the analysis of apposition. In case more than one-quarter of the analysed frame circumference was not visible due to insufficient flush or out of zoom, it was considered not analyzable and excluded. Likewise, if above one-third of the total stent length was not analyzable, pullbacks were excluded.

### OCT definitions

The OCT struts analyses applied previously reported definitions [14, 21, 23]. In brief, neointima area was defined as the tissue between the luminal border and the endoluminal border of the struts.

Strut coverage thickness was defined as the distance between the endoluminal side of the strut in the midpoint of its long axis and the intersection of the lumen contour with the straight line between the endoluminal side of the strut and the centre of gravity of the vessel. These measurements of the strut coverage, presented as a mean neointimal thickness, were performed on every visible stent strut twice. Struts were considered uncovered in case of a partial or complete absence of tissue coverage.

Strut apposition was evaluated strut by strut by measuring the distance between the center of the endoluminal strut border and the intersection between lumen contour and the line connecting the center of the endoluminal strut side and the gravitational center of the vessel. Strut malapposition was defined as a distance > 160 µm based on the consensus derived from the strut thickness of SES (153 m) and PES (148 m) plus the minimal axial resolution of OCT. Such an approach allowed for a blinded assessment. Strut protrusion was characterized by strut extension into the lumen for more than 160 µm, however, without clear separation from the vessel wall.

To account for a potential clustering of unfavourable strut outcome within the lesion, we also performed a lesion-level analysis for identification of stents with at least 5 or 10% of uncovered, malapposed and/or protruding struts.

In addition, the exploratory analysis of patterns of neointima thickness and strut coverage changes was performed. Neointima thickness was considered stable if the difference of neointima thickness between 3- and 9-year assessment was below 10 µm. The incidence of stents with increased, decreased and stable neointima thickness as well as increased, decreased and stable number of uncovered struts was reported.

### Quantitative coronary angiography

The 3- and 9-year coronary angiography was performed with the same angiographic projections as the baseline procedure. Off-line qualitative and quantitative coronary angiography (QCA) analysis was performed using the Cardiovascular Angiography Analysis System 5.11.1 (Pie Medical Imaging Systems, Maastricht, Netherlands), by an independent core laboratory [KCRI, Krakow, Poland].

The following angiographic parameters were calculated: minimum luminal diameter (MLD), percent diameter stenosis (%DS), reference vessel diameter (RVD), and a late lumen loss was defined as the difference between the 3- and 9-year MLD.

### Study endpoints

The primary study endpoint was defined as a change in the neointimal thickness evaluated by OCT between 3 and 9 years of follow-up. Secondary endpoints involved change in the percentage of uncovered, mallaposed and protruding struts, malapposition distance between the two OCT timepoints. In addition, the standard areas and volumes were reported. Finally, all-cause mortality and rates of target lesions revascularization (TLR) and ST classified according to the Academic Research Consortium (ARC) were reported until 9 years after the index procedure [24].

### Ethics

The study was approved by the local research ethics committee and was conducted in accordance with Declaration of Helsinki. All patients provided a written informed consent. At 9 years, patients were asked for a re-consent.

### Statistical analysis

Categorical variables are presented as counts and percentages, continuous variables as mean ± standard deviation. The unpaired and paired (serial analysis) t test, the chi square test (or Fishers’ exact test) and the Wilcoxon test were used for comparison of means and percentages. OCT intra- and inter-observer variability in the analysis of lumen area and stent area were assessed calculating the mean relative differences (as a percentage) with the standard deviation. All statistical tests were two-sided and the p value of 0.05 was considered statistically significant. Statistical analyses were performed using SPSS, version 21 (IL, US).

## Results

Of 156 consecutive SCAD patients who underwent PCI with implantation of the first-generation DES, 47 patients (22 SES and 25 PES) met the inclusion criteria and were assessed with OCT at 3 years post index procedure. Eight patients were excluded (insufficient pullback quality in six patients, OCT not feasible in two patients). Over the 6-year observation four patients died (two subjects due to cancer, one subject due to stroke, and one subject due to unknown reason, classified as probable ST according to ARC definition), six refused to participate in OCT follow-up and a contact was lost for five individuals. Two patients were excluded due to TLR with implantation of the second-generation DES.

**Fig. 1:**
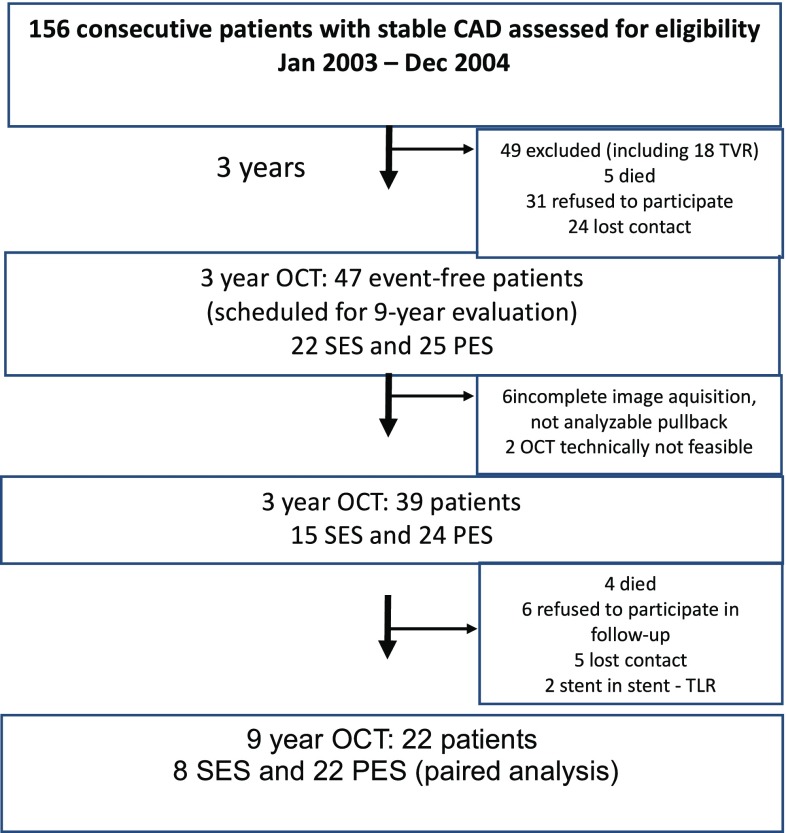
The flow chart of the study. *SES* sirolimus-eluting stent, *PES* paclitaxel-eluting stent, *CAD* coronary artery disease, *OCT* optical coherence tomography

Finally, a total of 22 sets of OCT pullback (8 SES and 14 PES) were evaluated in a paired OCT analysis between 3 and 9 years post procedure (Fig. 1).

Baseline clinical characteristics were well balanced between the SES and PES group (Table 1). In the PES arm a trend towards larger stent diameter (3.02 ± 0.27 vs. 3.12 ± 0.38 mm, p = 0.061) implanted in coronary arteries with larger RVD (2.57 ± 0.35 vs. 2.95 ± 0.44 mm, p = 0.006) was observed. The procedural and angiographic data have been presented in Tables 1, 2 and Suppl. Table 1.

**Table 1:** Baseline clinical characteristics

	SES (n = 22)	PES (n = 25)	p
Age (years)	60.4 ± 10.1	62.4 ± 9.6	0.802
Gender (male)	13 (59.1)	20 (80.0)	0.766
Previous MI	6 (27.3)	7 (28.0)	0.788
Hypertension	12 (54.5)	15 (60.0)	0.578
Hyperlipidemia	13 (59.1)	21 (84.0)	0.345
Diabetes mellitus	6 (27.3)	8 (32.0)	0.588
Obesity^a^	5 (22.7)	5 (25.0)	0.797
BMI (kg/m^2^)	27.3 ± 3.44	27.1 ± 3.24	0.732
Metabolic syndrome	4 (18.2)	5 (20.0)	0.874
Waist circumference	90 ± 11	76 ± 13	0.798
Total cholesterol (mg/dL)	163.0 ± 48.0	152.0 ± 41.7	0.743
LDL (mg/dL)	91.7 ± 44.8	86.0 ± 36.5	0.712
Smoker	3 (13.6)	3 (12.0)	0.674
Chronic kidney disease	3 (13.6)	3 (12.0)	0.654
Family history of cardiovascular disease^b^	9 (40.9)	11 (44.0)	0.831
LVEF (%)	52.2 ± 6.1	55.4 ± 7.3	0.537
DAPT for complete 12 months post PCI	20 (90.1)	22 (88.0)	0.732
Statin therapy	19 (86.4)	21 (84.0)	0.673
Treated vessel			
LAD	13 (59.1)	13 (52.0)	0.761
LCX	5 (22.7)	5 (20.0)	0.442
RCA	4 (18.2)	7 (28.0)	0.553
Lesion type (AHA/ACC)			
A	3 (13.6)3	0 (0.0)	0.432
B1	(13.6)	4 (16.0)	0.861
B2	7 (31.8)	8 (36.0)	0.891
C	9 (41.0)	13 (52.0)	0.522
Procedural characteristics			
Mean stent diameter (mm)	3.02 ± 0.27	3.12 ± 0.38	0.061
Mean stent length (mm)	24.47 ± 9.68	23.37 ± 7.11	0.781
Direct stenting	2 (9.1)	3 (12.0)	0.782
Post-dilatation	18 (81.8)	21 (84.0)	0.721
Max. inflation press. (atm.)	16.8 ± 2.6	16.2 ± 3.5	0.653
Obesity*	( *5 22.7*)	( *5 25.0*)	*0.797*
Smoker	( *3 13.6*)	(*3 12.0*)	*0.674*

*MI* myocardial infarction, *LVEF* left ventricle ejection fraction, *SES* sirolimus-eluting stent, *PES* paclitaxel-eluting stent, *atm*. atmospheres. Data are presented as mean ± standard deviation or count and proportion

^a^Body mass index (BMI) > 30

^b^Family history including of stroke, coronary artery disease and peripheral artery disease

**Table 2:** Paired optical coherence tomography and quantitative coronary angiography analysis between 3 and 9 years post implantation

	SES 3 years (n = 8)		SES 9 years (n = 8)			p	PES 3 years (n = 14)		PES 9 years (n = 14)		p
Lesion level and cross-sectional level analysis											
Lumen area (mm)	4.95 ± 1.29		4.76 ± 1.61			0.461	6.79 ± 1.91		6.39 ± 1.94		0.153
Minimal lumen area (mm^2^)	3.60 ± 0.95		3.22 ± 1.44			0.148	4.83 ± 1.86		4.53 ± 1.66		0.261
Lumen volume (mm^3^)	114.70 ± 59.15		120.48 ± 56.33			0.383	182.28 ± 59.66		178.83 ± 71.55		0.715
Stent area (mm^2^)	5.70 ± 0.98		5.78 ± 1.09			0.547	7.91 ± 1.91		7.52 ± 1.79		0.241
Minimal stent area (mm^2^)	4.49 ± 0.87		4.46 ± 1.00			0.547	6.18 ± 1.51		5.78 ± 1.53		0.241
Stent volume (mm^3^)	129.63 ± 59.37		141.03 ± 49.45			0.195	213.49 ± 59.99		208.26 ± 71.85		0.542
Neointima thickness, mm	0.10 ± 0.08		0.15 ± 0.17			0.195	0.13 ± 0.10		0.14 ± 0.12		0.952
Neointima area (mm^2^)	0.75 ± 0.54		1.07 ± 1.17			0.250	1.13 ± 0.79		1.14 ± 0.94		0.952
Min neointima area mm^2^	0.28 ± 0.26		0.39 ± 0.76			0.560	0.35 ± 0.35		0.18 ± 0.45		0.982
ISA (mm^2^)	− 0.01 ± 0.00 (n = 1)		0.14 ± 0.22 (n = 3)			0.250	0.03 ± 0.05 (n = 2)		0.05 ± 0.01 (n = 2)		1.000
Malapposed distance (mm)	0.15 ± 0.02 (n = 2)		0.21 ± 0.09 (n = 4)			1.000	0.31 ± 0.19 (n = 2)		0.14 ± 0.09 (n = 4)		1.000
Strut level analysis											
Total number of analyzed struts	1608		1615			0.742	2912		2929		0.856
Uncovered struts (%)	2.5 (0.01–28.3)		1.6 (0.01–5.4)			0.688	0.6 (0.01–3.0)		0.2 (0.01–4.7)		0.791
Malapposed struts (%)	0.002 (0.001–0.3)		0.6 (0.01–1.0)			0.125	0.0 (0.0–0.0)		0.01 (0.001–3.1)		0.625
Protruding struts (%)	1.2 (0.01–6.9)		0.0 (0.0–0.0)			0.250	0.001 (0.001–0.3)		0.0 (0.0–0.0)		0.250
Lesion level analysis (accounting for potential clustering effect)											
> 5% uncovered struts per stent		2 (25.0)		1 (12.5)	0.564			3 (21.4)	2 (14.3)		0.317
> 10% uncovered struts per stent		2 (25.0)		1 (12.5)	0.564			1 (7.1)	1 (7.1)		1.000
> 5% malapposed struts per stent		0 (0.0)		0 (0.0)	1.000			1 (7.1)	0 (0.0)		1.000
> 10% malapposed struts per stent		0 (0.0)		0 (0.0)	1.000			0 (0.0)	0 (0.0)		1.000
> 5% uncovered struts and > 5% malapposed struts per stent		0 (0.0)		0 (0.0)	1.000			0 (0.0)	0 (0.0)		1.000
Quantitative coronary angiography analysis											
Reference vessel diameter (mm)		2.48 ± 0.30		2.44 ± 0.36	0.250			3.03 ± 0.42	2.99 ± 0.28		0.214
Minimal luminal diameter (mm)		2.32 ± 0.34		2.05 ± 0.73	0.109			2.60 ± 0.57	2.51 ± 0.51		0.224
Diameter stenosis (%)		6.6 ± 7.0		18.6 ± 24.5	0.250			14.1 ± 15.0	16.5 ± 13.1		0.457
Late lumen loss 9–3 years		–		− 0.27 ± 0.52	–			–	− 0.10 ± 0.30		–
Binary restenosis n (%)		0 (0.0)		1 (12.5)	1.000			1 (7.1)	0 (0.0)		1.000

Data are presented as mean ± standard deviation or count and proportion

*SES* sirolimus-eluting stent, *PES* paclitaxel-eluting stent

Between 3 and 9 years following the index procedure a paired QCA analysis revealed similar MLD decrease in both subgroups (p = 0.561), with a late lumen loss of − 0.27 ± 0.52 mm in SES (p = 0.109) and − 0.10 ± 0.30 mm in PES (p = 0.224) (Table 2).

### OCT analysis

In a paired analysis 1608 and 1615 SES struts (p = 0.742) and 2912 and 2929 PES struts (p = 0.856) were assessed at 3 and 9 years, respectively.

### Neointimal thickness and area

**Fig. 2:**
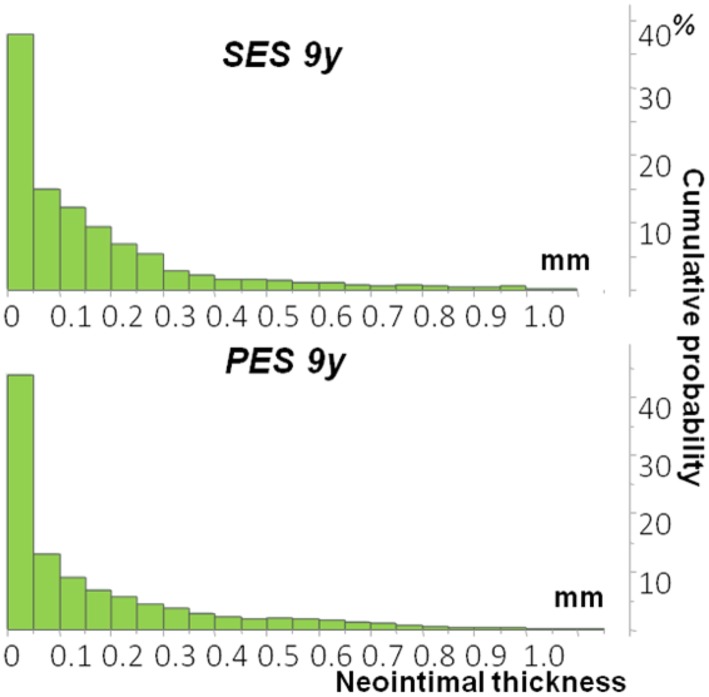
Cumulative distribution curves for neointima thickness (NIT) at 9 years post implantation. Data presented as median and interquartile range. *SES* sirolimus-eluting stent, *PES* paclitaxel-eluting stent

Between 3 and 9 years following stent implantation in a paired quantitative OCT analysis the neointimal thickness did not change significantly within SES [∆0.05 mm (− 0.03 to 0.14), p = 0.195] and PES group [∆0.01 mm (− 0.04 to 0.05), p = 0.951], which was also similar between both groups (p = 0.890) (Table 2). At 9 years some heterogeneity of neointimal response has been still observed in both SES and PES groups with the median neointimal thickness of 0.13 ± 0.10 mm in SES and 0.14 ± 0.12 mm in PES (p = 0.952) (Fig. 2). Detailed OCT measurements in SES and PES group at 3 and 9 years are presented in the Suppl. Table 2.

Over the six years of follow-up, the mean and minimal lumen and neointimal area did not change significantly with only numerical decrease of the lumen area [− 0.19 (− 0.55 to 0.17) mm vs. to 0.40 (− 0.87 to 0.08) mm, p = 0.460].

### Strut coverage apposition and protrusion

**Fig. 3:**
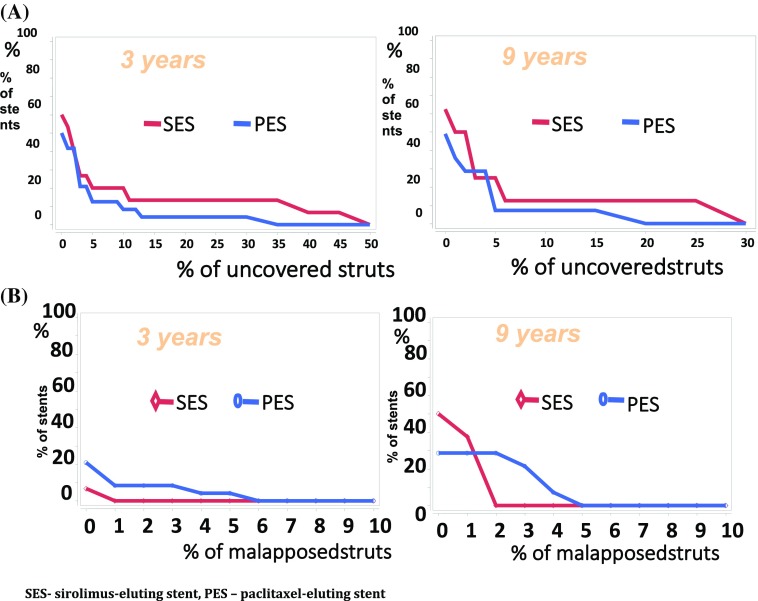
Cumulative distribution of uncovered struts (**A**) and mallaposed struts (**B**) at 3 and 9years post implantation. *SES* sirolimus-eluting stent, *PES* paclitaxel-eluting stent

There were no significant differences in terms of percentage of uncovered struts in either SES or PES over the 6 year observation period, with a stable median ratio of 2.5 at 3 years and 1.6% at 9 years in the SES (p = 0.688) and 0.6% at 3 years and 0.2% at 9 years in the PES (p = 0.791). The malapposition rates were low and remained stable (SES 0.002 vs. 0.6%, p = 0.125; PES: 0.0% vs. 0.01%, p = 0.625) (Table 2, Fig. 3). Likewise, protruding struts were rare in both SES- (1.2 vs. 0.0%, p = 0.250) and PES-treated (0.001 vs. 0.0%, p = 0.250) lesions.

Additionally, lesion-level analysis showed no difference in the proportion of lesions with > 5% (SES 25.0 vs 12.5%, p = 0.564; PES 21.4 vs. 14.3%, p = 1.000) and > 10% uncovered struts (SES 25.0 vs 12.5%, p = 0. 564; PES 7.1 vs. 7.1%, p = 1.000) in either SES or PES group between 3 and 9 years.

In the paired analysis, more than 5% of malaposed struts within the stent were noted in 7.1% of PES group at 3 years that were cured after 9 years from index procedure. No clustering effect of co-existing both > 5% malaposed and > 5% uncovered struts was noted within this study population (Table 2).

### Neointimal thickness and strut coverage change patterns

A paired analysis at the stent level revealed that 25.0 and 7.1% of SES and PES, respectively, demonstrated a stable neointima thickness over 6 year observation. In half of the cases in each group an increase exceeding 10 µm was reported, whereas 25.0% of patients in SES and 42.9% in PES presented with a redistribution of neointimal thickness and its decrease of more than 10 µm (Table 2).

Likewise, various types of strut coverage changes were observed in both SES and PES cases at the strut-to-strut analysis between 3 and 9 years, with stable number of uncovered struts observed among 25.0 and 21.4% of SES and PES patients respectively, and 35.5% of SES and 42.9% of PES presented with any decrease in the number of uncovered struts. Interestingly, in more than a third of patients (SES 37.5 vs. PES 35.7%) at least one additional strut per stent was identified as uncovered at 9 years compared to 3-year assessment (Suppl. Figures 1–4).

### Inter- and intraobserver variability

The quality of the measurements was confirmed by a low inter- and intraobserver variability, calculated for lumen and stent area. For inter-observer variability, the mean relative difference was 0.16 ± 4.65% for lumen area and 0.27 ± 5.11% for stent area; for intra-observer variability, the mean relative difference was 0.51 ± 1.35% for lumen area and 0.23 ± 2.10% for stent area.

## Discussion

To our knowledge, this is the longest, serial OCT follow-up of patients treated with DES published to date. The principal findings of this vascular healing response analysis performed among stable CAD patients treated with PCI with early-generation SES or PES that were event-free at 3 years post index procedure, could be summarized as follows: (1) at 3 and 9 years a similar neointimal thickness was observed, potentially suggesting lack of the ‘very late catch-up’ phenomenon after either SES or PES implantation; (2) the lumen, neointimal and malapposition areas remained stable over the 6-year follow up both in SES and PES patients; (3) a similarly low, though still persistent, rates of strut malapposition, protrusion and uncoverage were observed at 3 and 9 years in both groups; (4) some stents presented a clustering of uncovered struts at 3 and 9 years.

### Neointimal growth

Despite a growing body of OCT-derived evidence on the early and mid-term vessel healing and strut endothelialisation after implantation of vast array of continuously developed durable or biodegradable polymer drug eluting stent platforms [25–29], the patterns of very long-term vessel healing after coronary angioplasty remains vastly underexplored in *in vivo* investigations. The longest OCT follow-up of the first-generation DES implantation published so far reached 5 years, while the studies with serial OCT imaging reported data up to 4 years post procedure [9, 12, 15, 16, 20], of which some suggested a continuous neointimal growth following first-generation DES implantation, referred to as *late catch up* phenomenon. The neointimal thickness in the late phase ranged between 110 and 136 µm by OCT [12, 16, 20]. The late neointimal response has been related to the continuous inflammatory stimuli from the non-degradable polymer, in the absence of antiproliferative drug elution [30].

Some long-term observations suggest an independent-of-age decrease in the annual risk of TLF and ST risk beyond 5 years after implantation of early generation DES [31]. Nevertheless, whether the ongoing neointimal growth persists beyond 4–5 years (very late phase) cannot be concluded based on the currently available evidence.

The present study suggests no late augmentation of tissue growth up to 9 years after early-generation DES implantation. This may indicate that neointimal growth reaches plateau after extensive proliferation in the mid-phase after the procedure. However, the ‘two-points’ assessment does not allow to draw firm conclusions.

Our findings, limited to highly pre-selected event-free population of small sample size, still demand confirmation in a larger study, preferably with a multimodality approach and adequate statistical power to conclude on the impact of late intravascular imaging findings on clinical outcomes, thus contributing to amendment of current clinical practice and more tailored secondary prevention algorithms.

It should be also underlined that the direct clinical applicability of our findings is restricted given that both first-generation SES and PES are no longer used in clinical practice. Nevertheless, we are obliged to continue to follow-up all patients in whom first generation DES have been placed, which may have a substantial impact on the schemes of secondary prevention in these groups, including dual antiplatelet therapy.

Importantly, it still remains to be established whether the currently best-in-class DES may be subject to the *late catch up* phenomenon or accelerated neoatherosclerosis at the very long-term post implantation. A better current DES performance would be promising given technological improvements including enhanced polymer biocompatibility, bioresorbable polymers, thinner strut platforms with less blood flow and endothelial shear stress disturbances with subsequent more rapid and complete endothelialization, as well as a reduced propensity to strut fracture, all of which may have reduced their thrombogenicity [32]. Nevertheless, the multimodality imaging studies on the long-term safety of stent platforms currently deemed as *best-in-class* coronary systems are highly anticipated, as all current devices still encounter some problems of persistent inflammation, inappropriate neointima formation, and neoatherosclerosis observed in the reports with up to 5 years of follow-up [9, 33–35]. Hence, the studies on the very long-term imaging follow up should be encouraged, especially in the context of relatively young age and long life expectancy of patients undergoing PCI with contemporary DES [31].

### Strut coverage apposition and protrusion

The so far pathologic studies in metallic stents demonstrated that impaired endothelialisation, with the surrogate of ratio of uncovered to total stent struts per histological section, constitutes the best predictor of ST [11]. Accrued over time rate of struts covered with neointima, especially in the early phase after angioplasty, has been reported for both SES and PES, though with a lesser evidence for the latter [12, 30]. In the study by Räber et al. the rate of uncovered struts after first-generation DES was estimated at 1–2% at 5 year follow-up [16]. Despite some methodologic differences in the previous OCT studies and different time frames of the analysis, precluding direct comparisons, the present study confirmed a comparable rate of struts without neointimal coverage with a numerical improvement over next 6 years, in both SES and PES treated patients. Interestingly, the rates of uncovered struts in present study seem somewhat smaller compared to the 4.1% of uncovered struts in everolimus-eluting stents (EES) at 5 years posti-implantation in the EXAMINATION study, though the EES were implanted in the setting of STEMI into the lesion with substantial necrotic core demonstrated to substantially affect strut endothelialization [9].

The majority of previous OCT studies to date assessed the overall incidence of uncovered struts without accounting for the potential accumulation of unfavourable strut outcome within a single stent. To address this issue, we performed a lesion-level analysis that indicated some clustering of uncovered struts at 3 and 9 years. Importantly, we did not identify any cases with a rate of uncovered struts > 30% that were associated with a ninefold increase of ST in the post-mortem studies [2, 11, 30]. A significant clustering of uncovered struts accounting for > 10% was revealed only in 2 SES and 1 PES at 3 years, which is comparable to previously reported rates by Räber [16]. Given the overall low rate of uncovered struts in the total population at both 3- and 9-year assessment, our data confirm heterogenous neointimal healing reposne, in line with previously published observations [2, 12, 16, 20].

Apart from impaired strut coverage with neointima, DES-associated inflammation and toxicity may lead to late positive (outward) remodelling causing a late-acquired malapposition, reported to increase the risk of late ST [20].

In the present analysis, a very low overall rate of mallaposed struts (< 1 strut per stent) and minor incomplete stent area was noted in both study groups, that remained stable all over the 9-year follow-up. This may suggest that in the very long-term follow-up the very late quired malapposition is not that prevalent concern after implantation of early-generation DES.

Likewise, the phenomenon of strut protrusion, revealed in only 1.2 and 0.0% of SES and PES stents at 3 years, respectively, was practically absent at 9 year evaluation.

Our findings are in line with previous studies that also suggested very low, though numerically higher malapposition rates for SES (1.2%) compared to PES (0.7%) [16]. Due to the lack of baseline OCT we could not differentiate whether the malapposed struts at 3 year follow-up were present at the time of the baseline angioplasty and persistent over time or malapposition was aquiered during follow-up. Nevertheless, an important value of the present investigation is a double OCT evaluation, bringing the first insights into the very late apposition patterns of DES.

Finally, while interpreting the results of the present investigation it also relevant to recall the previous studies employing angioscopy for evaluation of healing response after metallic stent implantation. Angioscopic observation through a decade revealed that neointima thickness at the SES-implanted segments varied from no coverage to full coverage even at 3 years after the implantation [36]. Occasionally stent struts were still barely exposed into vascular lumen even at 3 years after angioplasty with SES implantation [36, 37]. However, the neointima structure appeared more homogenous, compared to PES treated lesions [36, 38].

## Limitations

Although the current study provides novel information regarding the longitudinal very late vascular healing response up to 9 years after implantation of early generation DES, it has to be viewed at in the light of several limitations. First, given a small sample size and observed losses in follow up, the presented results need to be interpreted with caution and demand confirmation in larger clinical trials. Despite relatively large number of struts included in the analysis, the limited number of stents, small size of the study population, non-randomized character of the study and potential selection bias, do not allow for drawing solid conclusions. Second, the evaluated in the present investigation SES and PES platforms are no longer used in present clinical practice. Third, there were two different types of OCT utilized at 3- and 9-year follow-up, which could affect the evaluation of the corresponding frames, given the lower resolution of the first-generation TD-OCT. Fourth, complete strut malapposition differentiation as either persistent (already present at time of the index procedure) or acquired later during follow-up period was hampered due to absence of the OCT examination at baseline. Fifth, in the present investigation no qualitative assessment of neointima was performed. Finally, the *two time-point* assessment does not ensure conclusive description of the dynamics of neointimal response—whether it stabilizes at long term reaching plateau after 3 years post PCI or rather it is a sequence of neointimal hyperplasia and regression.

## Future directions

Although the current findings, once confirmed in larger trial, bring relevant mechanistic insight into the very long term response after early-generation DES, which may have impact for secondary prevention of patients after PCI with SES and PES implantation, the present findings cannot be extrapolated to the novel DES platforms. In particular EES were shown to provide reportedly better intermediate-term strut apposition and coverage than first-generation DES, bioresorbable vascular scaffolds, and BMS, constituting at present overall best available combination of healing with suppression of neointimal hyperplasia at 6–12 months [39]. However, given the paucity of reports on vessel response beyond 5 years post-implantation, the multimodality imaging studies providing data on the long-term safety of the stent platforms currently deemed as best-in-class coronary stent systems may be expected as a subject of further research.

The present study did not aim to elaborate on the long-term clinical outcomes after PCI with early generation DES implantation. Further studies emploing multimodality imaging approach, concentrated more on correlation between the surrogate imaging parameters, including those derived also from newly developed techniques such as polarization sensitive OCT, OCT tissue characterization, photoacoustic imaging or near infrared fluorescence molecular imaging, and adverse clinical endpoints, are warranted to potentially ameliorate the current clinical practice and further improve the outcomes of patients undergoing PCI [40, 41].

## Conclusions

At 3 and 9 years after PCI, implantation of early-generation SES and PES may be associated with similar neointimal thickness, strut coverage, malapposition and protrusion, as assessed by serial OCT examination among patients with uneventful follow-up at 3 years post procedure. Given the small size of the presented study, a judicious interpretation of our results is necessary. Further larger multimodality imaging studies with clinical outcome correlation and inclusion of patients treated with contemporary stent platforms are warranted to evaluate the very long-term vessel response after PCI with DES and its clinical implications.

## Electronic supplementary material

Below is the link to the electronic supplementary material.

Supplementary material 1 (PDF 179 KB)

Supplementary material 2 (PDF 296 KB)

Supplementary material 3 (PDF 4371 KB)

Supplementary material 4 (PDF 2867 KB)

Supplementary material 5 (DOCX 54 KB)

Supplementary material 6 (DOCX 117 KB)

## References

[1] Adriaenssens T, Joner M, Godschalk TC, Malik N, Alfonso F, Xhepa E, De Cock D, Komukai K, Tada T, Cuesta J, Sirbu V, Feldman LJ, Neumann FJ, Goodall AH, Heestermans T, Buysschaert I, Hlinomaz O, Belmans A, Desmet W, Ten Berg JM, Gershlick AH, Massberg S, Kastrati A, Guagliumi G, Byrne RA (2017). Prevention of late stent thrombosis by an interdisciplinary global european effort. Optical coherence tomography findings in patients with coronary stent thrombosis: a report of the PRESTIGE Consortium (prevention of late stent thrombosis by an interdisciplinary global european effort). Circulation.

[2] Nakazawa G, Finn AV, Joner M, Ladich E, Kutys R, Mont EK, Gold HK, Burke AP, Kolodgie FD, Virmani R (2008). Delayed arterial healing and increased late stent thrombosis at culprit sites after drug-eluting stent placement for acute myocardial infarction patients: an autopsy study. Circulation.

[3] Stettler C, Wandel S, Allemann S, Kastrati A, Morice MC, Schomig A, Pfisterer ME, Stone GW, Leon MB, de Lezo JS, Goy JJ, Park SJ, Sabate M, Suttorp MJ, Kelbaek H, Spaulding C, Menichelli M, Vermeersch P, Dirksen MT, Cervinka P, Petronio AS, Nordmann AJ, Diem P, Meier B, Zwahlen M, Reichenbach S, Trelle S, Windecker S, Juni P (2007). Outcomes associated with drug-eluting and bare-metal stents: a collaborative network meta-analysis. Lancet.

[4] Daemen J, Wenaweser P, Tsuchida K, Abrecht L, Vaina S, Morger C, Kukreja N, Juni P, Sianos G, Hellige G, van Domburg RT, Hess OM, Boersma E, Meier B, Windecker S, Serruys PW (2007). Early and late coronary stent thrombosis of sirolimus-eluting and paclitaxel-eluting stents in routine clinical practice: data from a large two-institutional cohort study. Lancet.

[5] Souteyrand G, Amabile N, Mangin L, Chabin X, Meneveau N, Cayla G, Vanzetto G, Barnay P, Trouillet C, Rioufol G, Range G, Teiger E, Delaunay R, Dubreuil O, Lhermusier T, Mulliez A, Levesque S, Belle L, Caussin C, Motreff P, Investigators P (2016). Mechanisms of stent thrombosis analysed by optical coherence tomography: insights from the national PESTO French registry. Eur Heart J.

[6] Taniwaki M, Radu MD, Zaugg S, Amabile N, Garcia-Garcia HM, Yamaji K, Jorgensen E, Kelbaek H, Pilgrim T, Caussin C, Zanchin T, Veugeois A, Abildgaard U, Juni P, Cook S, Koskinas KC, Windecker S, Raber L (2016). Mechanisms of very late drug-eluting stent thrombosis assessed by optical coherence tomography. Circulation.

[7] Park SJ, Kang SJ, Virmani R, Nakano M, Ueda Y (2012). In-stent neoatherosclerosis: a final common pathway of late stent failure. J Am Coll Cardiol.

[8] Kang SJ, Mintz GS, Akasaka T, Park DW, Lee JY, Kim WJ, Lee SW, Kim YH, Whan Lee C, Park SW, Park SJ (2011). Optical coherence tomographic analysis of in-stent neoatherosclerosis after drug-eluting stent implantation. Circulation.

[9] Gomez-Lara J, Brugaletta S, Jacobi F, Ortega-Paz L, Nato M, Roura G, Romaguera R, Ferreiro JL, Teruel L, Gracida M, Martin-Yuste V, Freixa X, Masotti M, Gomez-Hospital JA, Sabate M, Cequier A (2016). Five-year optical coherence tomography in patients with ST-segment-elevation myocardial infarction treated with bare-metal versus everolimus-eluting stents. Circ Cardiovasc Interv.

[10] Joner M, Finn AV, Farb A, Mont EK, Kolodgie FD, Ladich E, Kutys R, Skorija K, Gold HK, Virmani R (2006). Pathology of drug-eluting stents in humans: delayed healing and late thrombotic risk. J Am Coll Cardiol.

[11] Finn AV, Joner M, Nakazawa G, Kolodgie F, Newell J, John MC, Gold HK, Virmani R (2007). Pathological correlates of late drug-eluting stent thrombosis: strut coverage as a marker of endothelialization. Circulation.

[12] Takano M, Yamamoto M, Mizuno M, Murakami D, Inami T, Kimata N, Murai K, Kobayashi N, Okamatsu K, Ohba T, Seino Y, Mizuno K (2010). Late vascular responses from 2 to 4 years after implantation of sirolimus-eluting stents: serial observations by intracoronary optical coherence tomography. Circ Cardiovasc Interv.

[13] Nakazawa G, Otsuka F, Nakano M, Vorpahl M, Yazdani SK, Ladich E, Kolodgie FD, Finn AV, Virmani R (2011). The pathology of neoatherosclerosis in human coronary implants bare-metal and drug-eluting stents. J Am Coll Cardiol.

[14] Tearney GJ, Regar E, Akasaka T, Adriaenssens T, Barlis P, Bezerra HG, Bouma B, Bruining N, Cho JM, Chowdhary S, Costa MA, de Silva R, Dijkstra J, Di Mario C, Dudek D, Falk E, Feldman MD, Fitzgerald P, Garcia-Garcia HM, Gonzalo N, Granada JF, Guagliumi G, Holm NR, Honda Y, Ikeno F, Kawasaki M, Kochman J, Koltowski L, Kubo T, Kume T, Kyono H, Lam CC, Lamouche G, Lee DP, Leon MB, Maehara A, Manfrini O, Mintz GS, Mizuno K, Morel MA, Nadkarni S, Okura H, Otake H, Pietrasik A, Prati F, Raber L, Radu MD, Rieber J, Riga M, Rollins A, Rosenberg M, Sirbu V, Serruys PW, Shimada K, Shinke T, Shite J, Siegel E, Sonoda S, Suter M, Takarada S, Tanaka A, Terashima M, Thim T, Uemura S, Ughi GJ, van Beusekom HM, van der Steen AF, van Es GA, van Soest G, Virmani R, Waxman S, Weissman NJ, Weisz G (2012). International Working Group for Intravascular Optical Coherence. Consensus standards for acquisition, measurement, and reporting of intravascular optical coherence tomography studies: a report from the International Working Group for Intravascular Optical Coherence Tomography Standardization and Validation. J Am Coll Cardiol.

[15] Stettler R, Dijkstra J, Raber L, Torii R, Zhang YJ, Karanasos A, Liu S, Crake T, Hamshere S, Garcia-Garcia HM, Tenekecioglu E, Ozkor M, Windecker S, Serruys PW, Regar E, Mathur A, Bourantas CV (2017). Neointima and neoatherosclerotic characteristics in bare metal and first and second generation drug eluting stents in patients admitted with cardiovascular events attributed to stent failure: an optical coherence tomography study. EuroIntervention.

[16] Raber L, Baumgartner S, Garcia-Garcia HM, Kalesan B, Justiz J, Pilgrim T, Moschovitis A, Khattab AA, Buellesfeld L, Wenaweser P, Meier B, Serruys PW, Juni P, Windecker S (2012). Long-term vascular healing in response to sirolimus- and paclitaxel-eluting stents: an optical coherence tomography study. JACC Cardiovasc Interv.

[17] Song L, Mintz GS, Yin D, Yamamoto MH, Chin CY, Matsumura M, Fall K, Kirtane AJ, Parikh MA, Moses JW, Ali ZA, Shlofmitz RA, Maehara A (2017). Neoatherosclerosis assessed with optical coherence tomography in restenotic bare metal and first- and second-generation drug-eluting stents. Int J Cardiovasc Imaging.

[18] Nakamura D, Attizzani GF, Toma C, Sheth T, Wang W, Soud M, Aoun R, Tummala R, Leygerman M, Fares A, Mehanna E, Nishino S, Fung A, Costa MA, Bezerra HG (2016). Failure mechanisms and neoatherosclerosis patterns in very late drug-eluting and bare-metal stent thrombosis. Circ Cardiovasc Interv.

[19] Radu MD, Raber L, Kalesan B, Muramatsu T, Kelbaek H, Heo J, Jorgensen E, Helqvist S, Farooq V, Brugaletta S, Garcia-Garcia HM, Juni P, Saunamaki K, Windecker S, Serruys PW (2014). Coronary evaginations are associated with positive vessel remodelling and are nearly absent following implantation of newer-generation drug-eluting stents: an optical coherence tomography and intravascular ultrasound study. Eur Heart J.

[20] Nakamura D, Lee Y, Yoshimura T, Taniike M, Makino N, Kato H, Egami Y, Shutta R, Tanouchi J, Yamada Y, Hara M, Sakata Y, Hamasaki T, Nishino M (2014). Different serial changes in the neointimal condition of sirolimus-eluting stents and paclitaxel-eluting stents: an optical coherence tomographic study. EuroIntervention.

[21] Prati F, Guagliumi G, Mintz GS, Costa M, Regar E, Akasaka T, Barlis P, Tearney GJ, Jang IK, Arbustini E, Bezerra HG, Ozaki Y, Bruining N, Dudek D, Radu M, Erglis A, Motreff P, Alfonso F, Toutouzas K, Gonzalo N, Tamburino C, Adriaenssens T, Pinto F, Serruys PW, Di Mario C, Expert, ’, s OCTRD (2012). Expert review document part 2: methodology, terminology and clinical applications of optical coherence tomography for the assessment of interventional procedures. Eur Heart J.

[22] Raber L, Mintz GS, Koskinas KC, Johnson TW, Holm NR, Onuma Y, Radu MD, Joner M, Yu B, Jia H, Menevau N, de la Torre Hernandez JM, Escaned J, Hill J, Prati F, Colombo A, di Mario C, Regar E, Capodanno D, Wijns W, Byrne RA, Guagliumi G, Group ESCSD (2018). Clinical use of intracoronary imaging. Part 1: guidance and optimization of coronary interventions. An expert consensus document of the European Association of Percutaneous Cardiovascular Interventions: Endorsed by the Chinese Society of Cardiology. Eur Heart J.

[23] Raber L, Mintz GS, Koskinas KC, Johnson TW, Holm NR, Onuma Y, Radu MD, Joner M, Yu B, Jia H, Meneveau N, de la Torre Hernandez JM, Escaned J, Hill J, Prati F, Colombo A, Di Mario C, Regar E, Capodanno D, Wijns W, Byrne RA, Guagliumi G (2018). Clinical use of intracoronary imaging. Part 1: guidance and optimization of coronary interventions. An expert consensus document of the European Association of Percutaneous Cardiovascular Interventions. EuroIntervention.

[24] Cutlip DE, Windecker S, Mehran R, Boam A, Cohen DJ, van Es GA, Steg PG, Morel MA, Mauri L, Vranckx P, McFadden E, Lansky A, Hamon M, Krucoff MW, Serruys PW, Academic Research C (2007). Clinical end points in coronary stent trials: a case for standardized definitions. Circulation.

[25] Ohtani H, Kimura S, Sugiyama T, Hishikari K, Misawa T, Mizusawa M, Hayasaka K, Yamakami Y, Kojima K, Sagawa Y, Hikita H, Ashikaga T, Takahashi A, Isobe M (2017). Comparison of vascular responses after different types of second-generation drug-eluting stents implantation detected by optical coherence tomography. Int J Cardiovasc Imaging.

[26] Kuramitsu S, Kazuno Y, Sonoda S, Domei T, Jinnouchi H, Yamaji K, Soga Y, Shirai S, Ando K, Saito S (2016). Vascular response to bioresorbable polymer sirolimus-eluting stent vs. permanent polymer everolimus-eluting stent at 9-month follow-up: an optical coherence tomography sub-study from the CENTURY II trial. Eur Heart J Cardiovasc Imaging.

[27] Suwannasom P, Onuma Y, Benit E, Gach O, von Birgelen C, Hofma SH, Sotomi Y, Bo X, Zhang YJ, Gao R, Garcia-Garcia HM, Wykrzykowska JJ, de Winter RJ, Serruys PW (2016). Evaluation of vascular healing of polymer-free sirolimus-eluting stents in native coronary artery stenosis: a serial follow-up at three and six months with optical coherence tomography imaging. EuroIntervention.

[28] Lee SWL, Tam FCC, Chan KKW, Lam SCC, Kong SL, Shea CP, Wong MKL, Wong AYT, Yung ASY, Zhang LW, Lam YM, Mintz GS, Costa RA, Stoll HP, Maehara A (2018). Establishment of healing profile and neointimal transformation in the new polymer-free Biolimus A9-coated coronary stent by longitudinal sequential optical coherence tomography assessments: The EGO-BIOFREEDOM study. EuroIntervention.

[29] Jaguszewski M, Aloysius R, Wang W, Bezerra HG, Hill J, De Winter RJ, Karjalainen PP, Verheye S, Wijns W, Luscher TF, Joner M, Costa M, Landmesser U (2017). The REMEDEE-OCT study: an evaluation of the bioengineered COMBO dual-therapy CD34 antibody-covered sirolimus-eluting coronary stent compared with a cobalt-chromium everolimus-eluting stent in patients with acute coronary syndromes: insights from optical coherence tomography imaging analysis. JACC Cardiovasc Interv.

[30] Nakazawa G, Finn AV, Vorpahl M, Ladich ER, Kolodgie FD, Virmani R (2011). Coronary responses and differential mechanisms of late stent thrombosis attributed to first-generation sirolimus- and paclitaxel-eluting stents. J Am Coll Cardiol.

[31] Yamaji K, Raber L, Zanchin T, Spitzer E, Zanchin C, Pilgrim T, Stortecky S, Moschovitis A, Billinger M, Schonenberger C, Eberli F, Juni P, Luscher TF, Heg D, Windecker S (2016). Ten-year clinical outcomes of first-generation drug-eluting stents: the sirolimus-eluting vs. paclitaxel-eluting stents for coronary revascularization (SIRTAX) very late trial. Eur Heart J.

[32] Simard T, Hibbert B, Ramirez FD, Froeschl M, Chen YX, O’Brien ER (2014). The evolution of coronary stents: a brief review. Can J Cardiol.

[33] Ino Y, Kubo T, Tanaka A, Liu Y, Tanimoto T, Kitabata H, Shiono Y, Shimamura K, Orii M, Komukai K, Satogami K, Matsuo Y, Yamano T, Yamaguchi T, Hirata K, Imanishi T, Akasaka T (2015). Comparison of vascular response between everolimus-eluting stent and bare metal stent implantation in ST-segment elevation myocardial infarction assessed by optical coherence tomography. Eur Heart J Cardiovasc Imaging.

[34] Hofma SH, Smits PC, Brouwer J, Velders MA, van ‘t Hof AW, Quere M, de Vries CJ, van Boven AJ (2015). Long-term follow-up of second-generation everolimus-eluting stents versus first-generation sirolimus-eluting stents in acute myocardial infarction: three-year results of the XAMI trial. EuroIntervention.

[35] Hofma SH, Brouwer J, Velders MA, van’t Hof AW, Smits PC, Quere M, de Vries CJ, van Boven AJ (2012). Second-generation everolimus-eluting stents versus first-generation sirolimus-eluting stents in acute myocardial infarction. 1-year results of the randomized XAMI (XienceV Stent vs. Cypher Stent in Primary PCI for Acute Myocardial Infarction) trial. J Am Coll Cardiol.

[36] Shibuya M, Ishihara M (2016). Coronary angioscopy for the evaluation of vessel response after drug-eluting stent implantation. Circ J.

[37] Ichikawa M, Bando K, Kijima Y (2017). Angioscopic observation of extremely late arterial repair after intracoronary implantation of the first-generation sirolimus-eluting stents. Int J Cardiol.

[38] Nakayoshi T, Ueno T, Sasaki KI, Yokoyama S, Ohtsuka M, Mitsutake Y, Itaya N, Chibana H, Sasaki M, Ishimatsu T, Kimura T, Fukumoto Y (2016). Differential angioscopic findings of neointimal coverage among first-, second-, and next generation drug-eluting stents. Int J Cardiol.

[39] Iannaccone M, D’Ascenzo F, Templin C, Omede P, Montefusco A, Guagliumi G, Serruys PW, Di Mario C, Kochman J, Quadri G, Biondi-Zoccai G, Luscher TF, Moretti C, D’Amico M, Gaita F, Stone GW (2017). Optical coherence tomography evaluation of intermediate-term healing of different stent types: systemic review and meta-analysis. Eur Heart J Cardiovasc Imaging.

[40] Ochijewicz D, Tomaniak M, Koltowski L, Rdzanek A, Pietrasik A, Kochman J (2017). Intravascular imaging of coronary artery disease: recent progress and future directions. J Cardiovasc Med.

[41] Prati F, Romagnoli E, La Manna A, Burzotta F, Gatto L, Marco V, Fineschi M, Fabbiocchi F, Versaci F, Trani C, Tamburino C, Alfonso F, Mintz GS (2018). Long-term consequences of optical coherence tomography findings during percutaneous coronary intervention: the centro per la lotta contro l’infarto—optimization of percutaneous coronary intervention (cli-opci) late study. EuroIntervention.

