# Supplementary material for: A serial 3- and 9-year optical coherence tomography assessment of vascular healing response to sirolimus- and paclitaxel-eluting stents
Source: Int J Cardiovasc Imaging. 2018 Aug 30;35(1):9–21. doi: 10.1007/s10554-018-1437-7 (PMC6373305; doi:10.1007/s10554-018-1437-7)
Supplement: Supplementary file 1 — Supplementary material 1 (PDF 179 KB) [file 10554_2018_1437_MOESM1_ESM.pdf]

Suppl. Fig.1

The percentage of neointimal thickness change patterns between 3 and 9 years (A). The percentage of strut coverage change patterns between 3 and 9 years (B).

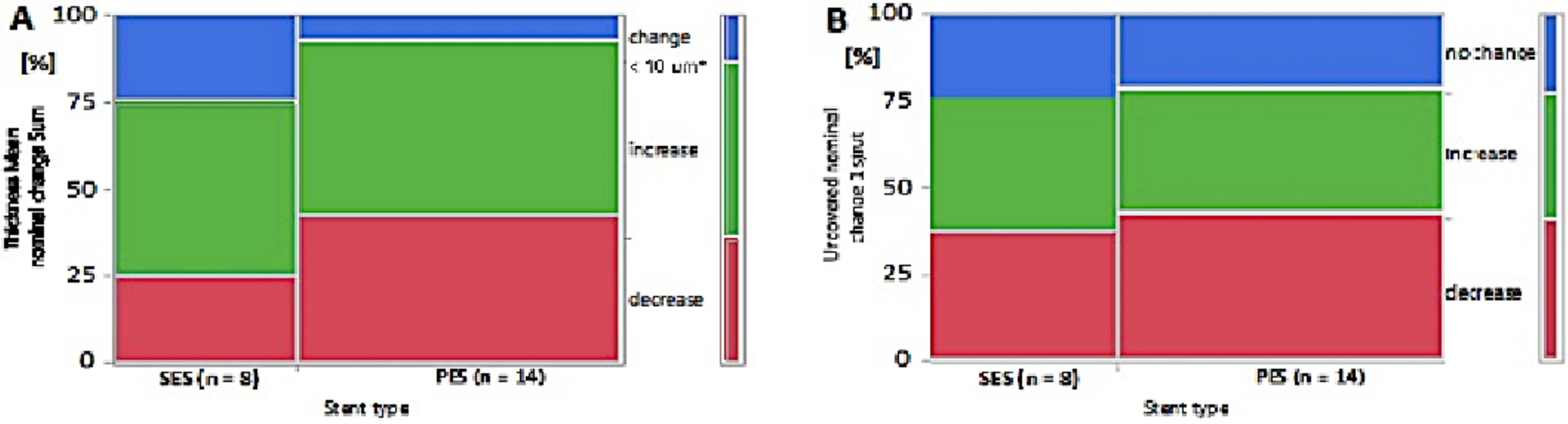

Contingency Analysis of Thickness Mean nominal change 10 um By Stent type
Mosaic Plot

Contingency Analysis of Uncovered nominal change 1 strut By Stent type
Mosaic Plot

*** The presented NIT change patterns need to be viewed at in the light of the thresholds of optical coherence tomography (OCT) resolution.**
SES- sirolimus-eluting stent, PES – paclitaxel-eluting stent
