# Supplementary material for: A serial 3- and 9-year optical coherence tomography assessment of vascular healing response to sirolimus- and paclitaxel-eluting stents
Source: Int J Cardiovasc Imaging. 2018 Aug 30;35(1):9–21. doi: 10.1007/s10554-018-1437-7 (PMC6373305; doi:10.1007/s10554-018-1437-7)
Supplement: Supplementary file 2 — Supplementary material 2 (PDF 296 KB) [file 10554_2018_1437_MOESM2_ESM.pdf]

Suppl. Fig. 2 Optical coherence tomography (OCT) documentation of strut coverage change patterns in a paired strut-to-strut analysis between 3 and 9years.

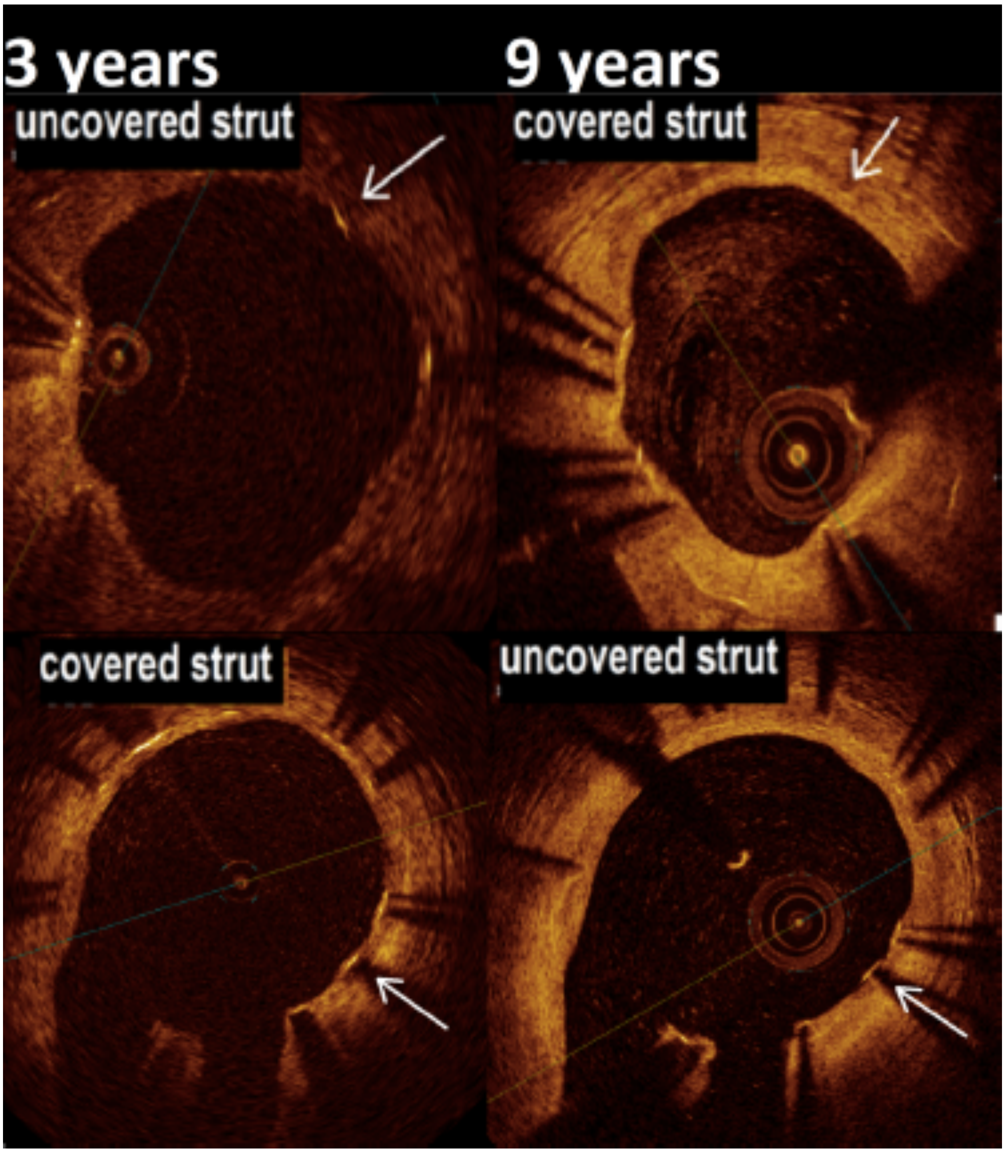

* The potential neointimal redistribution of neointima in the long – term leading to late uncoverage of some struts has to be viewed at in light of the threshold of optical coherence tomography (OCT) resolution.
SES- sirolimus-eluting stent, PES – paclitaxel-eluting stent
