# Supplementary material for: A serial 3- and 9-year optical coherence tomography assessment of vascular healing response to sirolimus- and paclitaxel-eluting stents
Source: Int J Cardiovasc Imaging. 2018 Aug 30;35(1):9–21. doi: 10.1007/s10554-018-1437-7 (PMC6373305; doi:10.1007/s10554-018-1437-7)
Supplement: Supplementary file 3 — Supplementary material 3 (PDF 4371 KB) [file 10554_2018_1437_MOESM3_ESM.pdf]

Suppl. Fig 3. **Spectrum of neointimal phenotypes at nine years follow up after implantation of SES (A) and PES (B) stents. Panels (A1, B1) show minimal coverage of stent struts with newly created neointimal tissue. Panels (A2, B2) show frames with maximal neointimal thickness.**

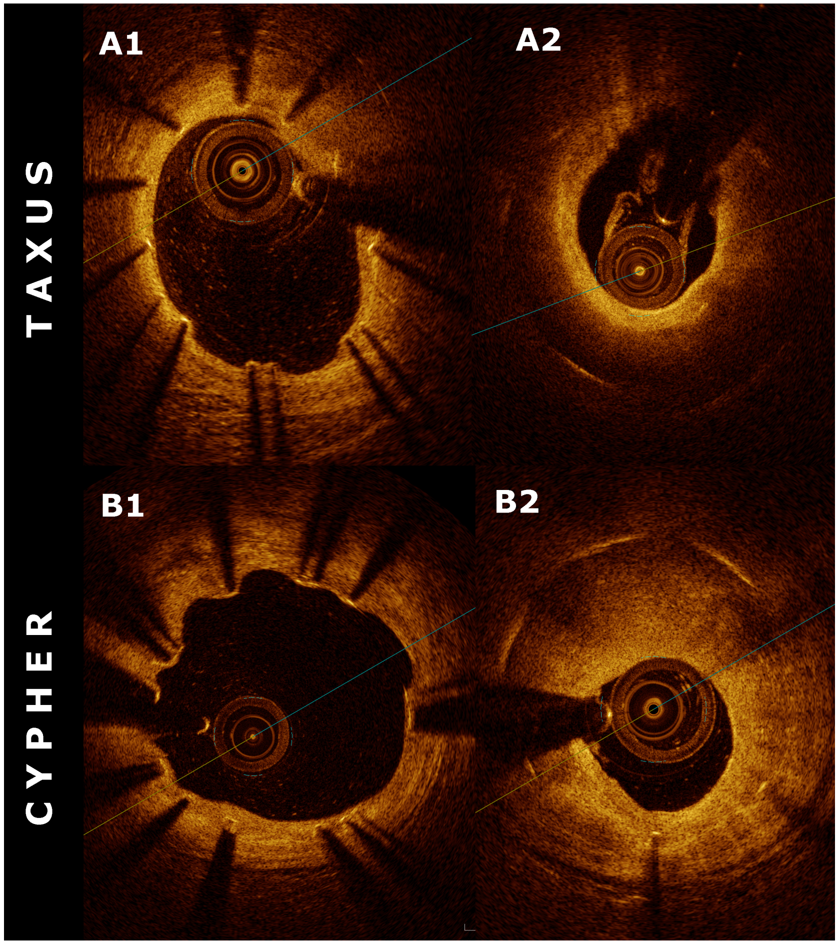

SES- sirolimus-eluting stent, PES – paclitaxel-eluting stent
