# Supplementary material for: A serial 3- and 9-year optical coherence tomography assessment of vascular healing response to sirolimus- and paclitaxel-eluting stents
Source: Int J Cardiovasc Imaging. 2018 Aug 30;35(1):9–21. doi: 10.1007/s10554-018-1437-7 (PMC6373305; doi:10.1007/s10554-018-1437-7)
Supplement: Supplementary file 4 — Supplementary material 4 (PDF 2867 KB) [file 10554_2018_1437_MOESM4_ESM.pdf]

Suppl. Fig 4.
Strut coverage patterns in the very long-term follow-up.

Optical coherent tomography (OCT) serial documentation of change in strut classification in different types of DES. Panels demonstrate tissue growth over PES (A1, B1) and SES (A2, B2) strut at 3- and 9-year OCT follow up. On the panels (A1, A2) the strut pointed by white arrow is classified as well-apposed uncovered strut, whereas white arrow on the panels (B1, B2) point at the same strut already covered in 9-years follow up.

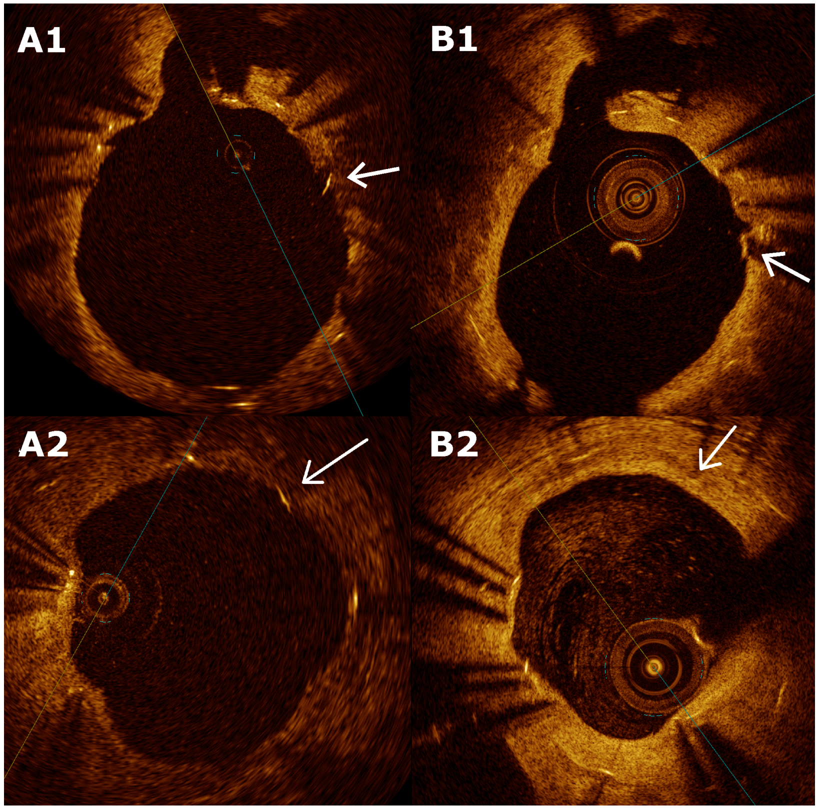

SES- sirolimus-eluting stent, PES – paclitaxel-eluting stent
