# Supplementary material for: A serial 3- and 9-year optical coherence tomography assessment of vascular healing response to sirolimus- and paclitaxel-eluting stents
Source: Int J Cardiovasc Imaging. 2018 Aug 30;35(1):9–21. doi: 10.1007/s10554-018-1437-7 (PMC6373305; doi:10.1007/s10554-018-1437-7)
Supplement: Supplementary file 5 — Supplementary material 5 (DOCX 54 KB) [file 10554_2018_1437_MOESM5_ESM.docx]

Supplementary **Table 1**

**Angiographic data at 3 years post implantation: comparison between the SES and PES groups.**

|  | **SES**  **(n=15)** | **PES**  **(n=24)** | **p** |
| --- | --- | --- | --- |
| **Minimal luminal diameter, mm** | 2.14 ± 0.43 | 2.51 ± 0.50 | 0.016 |
| **Diameter stenosis, %** | 15.20 ± 18.35 | 14.40 ± 14.13 | 0.665 |
| **Reference vessel diameter, mm** | 2.57 ± 0.35 | 2.95 ± 0.44 | 0.006 |
| **Binary restenosis, n (%)** | 1 (6.7) | 1 (4.2) | 0.615 |

Data are presented as mean ± standard deviation or count and proportion. SES- sirolimus-eluting stent, PES – paclitaxel-eluting stent
