# Supplementary material for: A serial 3- and 9-year optical coherence tomography assessment of vascular healing response to sirolimus- and paclitaxel-eluting stents
Source: Int J Cardiovasc Imaging. 2018 Aug 30;35(1):9–21. doi: 10.1007/s10554-018-1437-7 (PMC6373305; doi:10.1007/s10554-018-1437-7)
Supplement: Supplementary file 6 — Supplementary material 6 (DOCX 117 KB) [file 10554_2018_1437_MOESM6_ESM.docx]

**Supplementary Table 2.**

**Optical coherence tomography (OCT) comparison of SES and PES groups at 3 and 9 years post implantation.**

| **Parameter** | **3 years** | | | **9 years** | | |
| --- | --- | --- | --- | --- | --- | --- |
|  | **SES**  **(n=15)** | **PES**  **(n=24)** | **p** | **SES**  **(n=8)** | **PES**  **(n=14)** | **p** |
| **Lumen area, mm** | 4.77 ± 1.43 | 6.54 ± 1.88 | 0.005 | 4.76 ± 1.61 | 6.39 ± 1.94 | 0.065 |
| **Minimal lumen area, mm^2^** | 3.09 ±1.26 | 4.78 ± 1.89 | 0.005 | 3.22 ± 1.44 | 4.53 ± 1.66 | 0.088 |
| **Lumen volume, mm^3^** | 121.01  ± 55.30 | 169.54  ± 60.35 | 0.023 | 120.48  ± 56.33 | 178.83 ± 71.55 | 0.041 |
| **Stent area, mm^2^** | 5.97 ± 1.31 | 7.52 ± 1.87 | 0.021 | 5.78 ± 1.09 | 7.52 ± 1.79 | 0.020 |
| **Minimal stent area, mm^2^** | 4.64 ± 1.23 | 5.95 ± 1.64 | 0.014 | 4.46 ± 1.00 | 5.78 ± 1.53 | 0.044 |
| **Stent volume, mm^3^** | 150.42  ± 60.78 | 195.85  ± 66.82 | 0.040 | 141.03 ± 49.45 | 208.26  ± 71.85 | 0.020 |
| **Neointima thickness, mm** | 0.16 ± 0.12 | 0.11 ± 0.09 | 0.145 | 0.15 ± 0.17 | 0.14 ± 0.12 | 0.838 |
| **Neointima area, mm^2^** | 1.21 ± 0.85 | 0.99 ± 0.71 | 0.371 | 1.07 ± 1.17 | 1.14 ± 0.94 | 0.785 |
| **ISA, mm^2^** | 0.08 ± 0.00  (n=1) | 0.05 ± 0.02 (n=4) | 0.157 | 0.14 ± 0.22  (n=3) | 0.05 ± 0.01  (n=2) | 0.567 |
| **Malapposed distance, mm** | 0.14 ± 0.02  (n=2) | 0.18 ± 0.14  (n=7) | 0.770 | 0.21 ± 0.09  (n=4) | 0.14 ± 0.09  (n=4) | 0.149 |
| **Uncovered struts per stent, %** | 1.7  (0.0 – 5.4) | 0.7  (0.0 – 3.3) | 0.391 | 1.6  (0.0 – 5.4) | 0.2  (0.0 - 4.7) | 0.452 |
| **Malapposed struts per stent, %** | 0.0  (0.0 – 0.0) | 0.0  (0.0 – 0.3) | 0.288 | 0.6  (0.0 – 2.0) | 0.0  (0.0 – 3.1) | 0.752 |
| **Protruding struts per stent, %** | 0.0  (0.0 – 5.3) | 0.0  (0.0 – 0.6) | 0.114 | 0.0  (0.0 – 0.0) | 0.0  (0.0 – 0.0) | 0.186 |
| **>5% uncovered struts per stent** | 4 (26.3) | 3 (12.5) | 0.396 | 2 (25.0) | 3 (21.4) | 0.620 |
| **> 10% uncovered struts per stent** | 3 (20.0) | 2 (8.3) | 0.354 | 1 (12.5) | 1 (7.1) | 0.606 |
| **>5% malapposed struts per stent** | 0 (0.0) | 1 (4.2) | 1.000 | 0 (0.0) | 0 (0.0) | 1.000 |
| **>10% malapposed struts per stent** | 0 (0.0) | 0 (0.0) | 1.000 | 0 (0.0) | 0 (0.0) | 1.000 |
| **>5% uncovered struts per stent and >5% malapposed struts per stent** | 0 (0.0) | 0 (0.0) | 1.000 | 0 (0.0) | 0 (0.0) | 1.000 |

Data are presented as mean ± standard deviation, median and interquartile range or count and proportion. SES- sirolimus-eluting stent, PES – paclitaxel-eluting stent
